# Criminal governance in a large European city: The case of gangs in London

**DOI:** 10.1177/14773708251315581

**Published:** 2025-03-31

**Authors:** Paolo Campana, Federico Varese, Cecilia Meneghini

**Affiliations:** 2152 University of Cambridge, UK; SciencesPo Paris, France; 3286 University of Exeter, UK

**Keywords:** Access to services, communities, gangs, governance, residential stability, socioeconomic disadvantage

## Abstract

This study explores criminal governance among urban gangs operating in a large European city. It does so by introducing the Crim-Gov questionnaire, a novel survey tool purposely designed to systematically capture key governance-related dimensions of criminal groups’ activities, and applying it to the case of London. It identifies instances of criminal governance among gangs operating in the city and shows that governance activities are only carried out by a subset of groups, thus suggesting that a more complex set of skills is required to provide criminal governance, particularly beyond illegal markets. Governance-type gangs are spatially clustered and impact 4.7% of all London communities. Next, the paper explores the neighbourhood structural conditions associated with governance and non-governance gang presence. It shows that, while socioeconomic disadvantage increases *all* types of gang presence, barriers to local services, including affordable housing, only increase the probability of governance-type gangs. Criminal governance is more likely to emerge when services provided by local and central authorities are of lesser quality. Results also indicate a negative association between residential mobility and criminal governance, suggesting that a certain degree of residential stability is needed for criminal governance to emerge. These findings point to the importance of separating different types of gang activities, lending support for a broader theory that situates (some) urban gangs within the spectrum of organised crime governance. It is also crucial for law enforcement and local stakeholders to identify governance-type gangs and treat them separately from non-governance ones. When developing interventions to tackle the emergence and persistence of governance-type gangs, particular emphasis should be placed on improving access to and quality of public services.

## Introduction

Criminal groups operating in illegal markets are often lumped together under broad labels such as ‘organised crime’ or ‘gang’. And yet such groups engage in a variety of activities. Some *produce* illegal goods and services, such as cocaine in Colombia or cyber fraud online. Others specialise in *trading* goods from one place to another, such as drug traffickers. It is now well accepted that a third type of group goes beyond production and trade and attempts to *govern* communities by playing a role in their life, as well as extort/protect legal businesses and influence the decisions of politicians and public officials (Campana and Varese, 2018).

Criminal governance should be thought of as a continuum. Some groups have taken over state functions, such as imposing restrictions on people's behaviour, enforcing agreements, solving disputes, and enforcing a rough kind of justice. Others have made only limited inroads into these areas of social life, yet display signs that their activities go beyond ordinary crime (Varese, 2010). Starting with the pioneering work of Landesco (1929), notable studies have documented how traditional mafias, Latin American ‘cartels’ and insurgencies take over key state functions (Gambetta, 1993; Varese, 2001; Chu, 2000; Hill, 2003; Uribe et al., 2022; Arjona et al., 2015). At the other end of the governance spectrum, scholars have shown that gangs are able to influence communities in settings such as Medellin (Gordon, 2020), London and Nottingham (Densley, 2013: 61–62; Fellstrom, 2008), while they have not become an alternative government. Despite variations in degree, criminal groups across a wide geographical spectrum can go beyond illegal production and trade and involve themselves in imposing a degree of order on the communities where they nest.

The aim of this paper is twofold: first, to explore the extent to which urban gangs in a large European city are involved in more than just selling illegal products such as drugs; and second, to investigate the neighbourhood structural conditions associated with such gang presence. To do so, we first need to establish whether gangs engage in racketeering, influence business decisions, have a degree of control over local officials, and are involved in community activities. If the answer to such question is positive, we define such gangs as ‘governance-type’, while acknowledging that their behaviour is not quite on a par with traditional mafias. To identify such gangs, we introduce a novel instrument, the Crim-Gov questionnaire. We submit the instrument to police officers tasked with policing gangs in London.

We then leverage the data collected through the expert survey to explore the following questions: What are the structural conditions under which governance (and non-governance) gangs are present in communities? And are there differences between governance and non-governance gangs?

Theoretically, this paper builds on two separate strands of research. First, it expands on authors who have examined the governance dimension of urban gangs in the United States and the Global South, especially in Latin America and the Caribbean. Such studies are rare in Europe, and they tend to be qualitative. While qualitative studies are most insightful in offering deep accounts of local dynamics, our approach in this paper is best suited to explain why governance-type gangs are present in some neighbourhoods and not in others. In short, this paper contends that it is imperative to distinguish governance from other gangs’ activities. Second, it connects the study of governance-type gangs with the broader gang literature exploring the *structural* conditions that might lead to the emergence and persistence of gangs in local communities (see Decker et al., 2022: 36–66 for a review and a most useful taxonomy of theories; due to the nature of our data, in this work we leave aside any consideration on ‘culture’ and focus exclusively on elements of ‘structure’). We expand on US-based works by offering novel insights from a large urban city in Europe. In addition, by integrating such insights with our criminal governance perspective, we will explore whether we can identify any difference in structural characteristics that lead to the presence of the two distinct types of gangs: governance and non-governance.

The text continues as follows. The second section discusses criminal governance, while the third section offers a theoretically informed discussion of the neighbourhood-level structural factors potentially conducive to the presence of (governance and non-governance) gangs in a local area. The fourth section then introduces the data and methods we use. The fifth section presents the results of our expert survey on the presence/absence of criminal governance in London based on the purposely developed Crim-Gov questionnaire. The sixth section tests the factors conducive to the presence of gangs in London neighbourhoods, separating those involved in criminal governance from non-criminal governance ones. The seventh section concludes.

## Criminal governance

Criminal governance can impact both legal and illegal markets, as well as communities more broadly. In illegal markets, criminal governance often involves protection against competition, enforcement of cartel agreements, dispute resolution, labour racketeering, intimidation of lawful right-holders, protection against theft and police harassment, informal credit and debt recovery, and defence against extortionists (see Varese, 2014 for a review; Schelling, 1971 more generally on criminal governance). This form of governance has been documented in various traditional Mafia organisations, such as the Sicilian Mafia (Sabetti, 1984; Gambetta, 1993), the Italian-American Mafia (Reuter, 1983), the Russian Mafia (Varese, 2001), the Hong Kong Triads (Chu, 2000), the Japanese Yakuza (Hill, 2003), the Calabrian `Ndrangheta (Paoli, 2003; Varese, 2011), and the Neapolitan Camorra (Campana, 2011). Additionally, this phenomenon is increasingly observed in Latin America (Brophy, 2008; Locks, 2014; Lessing, 2021). Criminal governance of markets has also been identified in urban areas like Stockholm, Sweden (Skinnari et al., 2012), London, England (Levi, 1981), and Salford, England (Campana and Varese, 2018). Levi (2008: 62) notes that the Kray and Richardson gangs in London sought to monopolise extortion and other criminal activities.

Criminal governance can go beyond the control over markets and extend to broader community governance. An example is a small Sicilian village studied by Sabetti (1984), where distrust of ineffective central authority led to the rise of a criminal group headed by Calogero Vizzini. Vizzini provided protection to peasants against banditry and soon became one of the most powerful bosses of the Sicilian Mafia in the twentieth century. His success led villagers to approach him with other issues, transforming the mafia into a form of local self-government based on some degree of consent (Sabetti, 1984: 104). This type of community involvement is not restricted to traditional mafia territories. Lessing (2022: 11) observes that in urban peripheries worldwide, local criminal organisations often govern as much as, if not more than, the state. When asked, ‘Who is in charge here?’ the answer often points to a local gang, mara, militia, faction, collective, or cartel. Residents, police, politicians, and researchers recognise this reality (Lessing, 2022: 11). The 2020 Latino barometer survey found that in Latin America, 14% of respondents reported governance by a criminal or armed group in their neighbourhood, affecting between 77 and 101 million citizens (Uribe et al., 2022). This phenomenon is also relevant in prisons (Skarbek, 2014) and in urban peripheries in the Global North.

In conclusion, mafias and gangs can control illegal markets such as drugs and sexual services, but their influence can also extend into legal markets and community life. This phenomenon should be viewed as a continuum: in some settings, mafias take over state functions while, in others, their control over the legal economy and community is limited. Studying both ends of this spectrum is important, as one can evolve into the other. The first goal of this paper is to identify elements of criminal governance among urban gangs operating in London using a novel instrument, and separate such gangs from the non-governance ones.

## Explaining gang presence

The second goal of this paper is to explore which local area characteristics make a neighbourhood more vulnerable to governance (and non-governance) gang presence. The importance of structural conditions in understanding the emergence and persistence of gangs has a long history that can be traced back to the early works of Thrasher (1927) and Shaw and McKay (1942). Works in this tradition of gang studies have highlighted the role of a number of socio-economic factors that are associated with higher levels of gang presence: socio-economic disadvantage, immigrant concentration and residential mobility (Katz and Schnebly, 2011: 379; Pyrooz et al., 2010). While operationalisations vary across authors, this school of thought stemming from the Chicago School has interpreted such factors as indicators of social disorganisation within a community, with the idea that ‘these forms of structural disadvantage erode the foundations of normative authority and hinder the development of social cohesion and mutual trust’ (Katz and Schnebly, 2011: 379; see also Sampson and Groves, 1989; Bursik and Grasmick, 1993; Decker et al., 2022: 46–50 for a broader discussion). A competing theoretical explanation based on the idea of strain/anomie (Merton, 1938; Cloward and Ohlin, 1960; see Pyrooz et al., 2010: 869–871 and Decker et al., 2022: 55–58 for a broader discussion) also emphasises the key role of economic disadvantage in explaining gang presence at the neighbourhood level. The hypothesised mechanism, however, differs from social disorganisation and rests on the idea that people in ‘economically deprived areas experience more strains than those with high economic status’ (Pyrooz et al., 2010: 870). In line with Pyrooz et al. (2010: 874), our goal in this paper is not to test which competing theoretical approach best explains the case of gangs in London, but to offer a theoretically informed examination of gang presence in London neighbourhoods. We thus retain from both approaches the emphasis on neighbourhood-level socio-economic disadvantage, which we operationalise in our work using four different measures: income deprivation, household income, educational disadvantage and health deprivation. Poverty, income deprivation, low or no educational qualification and health deprivation are all part of what Sampson and Bean (2006) called ‘hard facts of life’. Empirical studies have indeed found a positive association between a community's socioeconomic disadvantage and gang presence (e.g., Curry and Spergel, 1988, focusing on gang homicides; Tita et al., 2005; Papachristos and Kirk, 2006; Katz and Schnebly, 2011; see also Decker et al., 2022: Ch. 2 for a broader discussion). Yet, none of these studies considered the differential impact that socioeconomic disadvantage might have on the *type* of gangs that are active in a community.

Social disorganisation theorists have also highlighted the role of migrants’ concentration on gang formation, as high levels of migrants’ concentration are considered to weaken social ties and hinder patterns of communication (Papachristos and Kirk, 2006; Sampson et al., 1997; Sampson and Groves, 1989; Shaw and McKay, 1942; see also Curry and Spergel, 1988). Additionally, migrant populations might feel marginalised and lack trust in official institutions, such as the police. In our work, we operationalise migrants’ concentration based on the proportion of residents not born in the United Kingdom.

Social disorganisation theorists have also suggested that (high) residential mobility can weaken social ties and mechanisms of control, thus leading to greater gang presence (Thrasher, 1927; see also Sampson et al., 1997; Sampson and Groves, 1989; Shaw and McKay, 1942). The evidence on this point, however, is far from conclusive. Studies from the United States have found a negative relationship between residential mobility and gangs (Wells and Weisheit, 2001; Katz and Schnebly, 2011; cf. Curry and Spergel, 1988, showing that high levels of gang *violence* are associated with unstable communities). Katz and Schnebly (2011: 377) found that gang membership (and not activity) ‘is less likely in social contexts characterized by either a residentially unstable population or rapidly changing structural conditions’ (it should be noted that they link gang members to neighbourhoods based on the residential address of members and not the area in which the gang operates; the two might not perfectly overlap). A certain degree of neighbourhood stability may thus be required for gangs to successfully operate, for example, by allowing them to gather information on the community (on the importance of information, see Reuter, 1983; Gambetta, 1993). The transmission of gang culture might also be hindered in neighbourhoods characterised by a rapidly changing residential composition (Katz and Schnebly, 2011). Studies so far have looked at the impact of residential mobility on gang presence, regardless of whether they were involved in criminal governance. For instance, residential instability might facilitate the presence of purely predatory gangs due to weakened social ties, but governance-type gangs might require some degree of community stability to exert their criminal rule. In this work, we consider a measure capturing the 5-year residential mobility in London neighbourhoods.

Additional factors explored in the gang literature relate to population density and population age structure. A higher population density is postulated to be associated with increased anonymity among community members, therefore conducive to lower informal social control (Katz and Schnebly, 2011: 380). A larger proportion of youth residents is associated with higher gang presence through various mechanisms. Social control theorists argue that a higher proportion of young people means a lower proportion of the adult population capable of exerting effective social control (Tita et al., 2005: 277; Katz and Schnebly, 2011). At the same time, age-crime curve analyses show that young people are more prone to crime in general (Farrington, 1986), and they can also constitute a larger reservoir for recruitment (Decker et al., 2022). We include both factors – population density and population age structure – in our analysis.

A separate strand of the literature has looked at the factors that might account for the emergence of criminal governance. Generally speaking, criminal governance is thought to emerge in a setting characterised by a weak state presence (Sabetti, 1984; Gambetta, 1993; Varese, 2001). Varese (2011) has documented how the emergence of new, unregulated markets generates incentives among entrepreneurs to create cartels, even with the use of criminal groups. Looking at neighbourhoods in Medellín, Colombia, Blattman et al. (2021) show that gangs fill the vacuum of state order yet do not completely crowd out state presence. In his ethnographic work on a housing estate in a large American city, Venkatesh (1997) illustrates how a local street gang started to provide services to the community, filling in the gap left by formal institutions, including the housing authority. In sum, a key factor highlighted in the criminal governance literature is the role played by the efficiency and effectiveness of the state (and other local authorities) *beyond* law enforcement activity (Campana and Varese, 2018). Such efficiency and effectiveness can be conceptualised in multiple ways. At the local (neighbourhood) level in a large city in the Global North, we proxy the efficiency and effectiveness of states (and local institutions) based on the accessibility to local services in a given area.

In conclusion, an influential stream of literature stemming from the early works of sociologists from the Chicago School has emphasised the importance of neighbourhoods – and their structural characteristics – in understanding gang presence. Scholars have highlighted the role of socio-economic disadvantage, migrants’ concentration and residential mobility. Yet, studies so far have mostly looked at the US context and they haven’t separated different types of gangs based on their activities. A separate stream of literature has emphasised the importance of criminal governance and the need to consider criminal groups, including gangs, involved in governance activities separately from the others. Such literature has also suggested that an ineffective state presence might be linked to the emergence of criminal governance. This paper brings together these two strands by first identifying whether criminal governance-type gangs exist in the case of London, and then testing whether the factors highlighted in both gang and criminal governance literature play a role in explaining the presence of both governance and non-governance gangs in London.

## Data and methods

### The Crim-Gov questionnaire

To identify instances of criminal governance at the group level, we employ a novel survey instrument purposely developed for this study: the Crim-Gov questionnaire (see Appendix 1 for the full questionnaire). It captures five dimensions of group activities: the ability (i) to generate fear in a community; (ii) to coerce legal businesses; (iii) to influence public officials; (iv) to control illegal market(s), but also (v) the group's involvement in community activities. The questionnaire has been widely discussed with practitioners across the United Kingdom and trialled in one police area before being rolled out in the entire jurisdiction of the London Metropolitan Police.

Each dimension of group activities is measured on a five-point scale, where 0 indicates that a group does not engage in that activity, and 4 indicates that a group is strongly involved in the activity.^1^ Examples are given for each dimension to provide police officers and analysts with an operational definition of the activities and place them in the London context. The level of fear generated by a group is assessed by considering how much ‘the group engages in disrespectful behaviour, threatens members of the public, resorts to violence, generates a “no grass” culture in the community’ (quote from the questionnaire). The coercion of legal businesses is exemplified as the ability to ‘threaten business owners, engage in extortion, influence business decisions’. The ability to influence public officials considers, for example, the extent to which ‘the group influences probation officers and other officials involved in its case file, influences local officials’ decisions, influences local councillors and politicians’. Illegal market(s)’ control is exemplified as the extent to which ‘the members sell illegal products, they decide who is allowed to sell illegal products, they successfully keep rival groups away from their territory’. Finally, a group's involvement in community activities considers to what extent the group ‘offers assistance to neighbourhood residents’ and ‘settles disputes between community members’.

As this paper focuses on the criminal governance dimension of gangs operating in London, we have decided to ask police officers/analysts assigned to a local gang unit to fill out the Crim-Gov questionnaire (this is akin to the strategy that Miller devised for the first National Youth Gang Survey using police as experts: Miller, 1992 [1982]: 10–16). Officers were asked to score the gang(s) for which they have close knowledge. We have not supplied officers with any pre-set definition of gang, but left it to their professional knowledge to identify the list of relevant groups based on intelligence and other police records.^2^

In the case of London, we adopted a multistage approach leveraging the institutional architecture of the London Metropolitan Police. Greater London is divided into 12 basic command units, which represent our first stage. The questionnaire was then trickled down to 32 specialist gang officers, one for each London Borough. These specialist gang officers were then tasked with filling in a questionnaire for each gang operating in their area in consultation with local specialist officers/analysts as well as available intelligence. Questionnaires were then gathered in an electronic format by our point of contact who cross-checked (unlikely) duplicate records, sanitised the data and then forwarded them in a fully anonymised form; neither gangs’ names nor any other information besides the responses to our questionnaire was shared with us. The identity of respondents was also kept confidential. The survey was administered between April and November 2019.

While it is possible to collect information directly from gang members, we opted for an alternative method based on a specific type of expert survey. This method allows us to systematically build a comprehensive picture of the presence and *absence* of gangs across a very large urban area, that is, Greater London, in a cost-effective and limited time-consuming manner. It comes, however, with its own limitations. Police officers might not be the best placed to provide the most accurate picture when it comes to communities’ attitudes, for example the level of fear generated by a gang; they might underestimate the impact of threats and coercion of legal business and community members, as these might be underreported due to the fear of reprisals; and they might be reluctant to report on the influence on public officials (some of whom might be fellow police officers; the fact that we only ask for a score and not further evidence might limit the impact of this bias but not eliminate it completely). Furthermore, there is always the possibility that a gang has not been identified by the police at the time of the survey. Additionally, our method is not able to elicit any evidence of cultural meanings associated with life in a gang or internal mechanisms. We acknowledge that specialised police officers are only one potential source of information on gangs (and criminal groups more generally) and that our instrument would provide a more comprehensive picture if the set of respondents is expanded in future works by adding other (non-police) officials and local stakeholders. In Table A1 in Appendix 2, we offer an external validity control of our survey instrument leveraging data from the Public Attitude Survey conducted by the Mayor of London, Office for Policing and Crime; survey data corroborate our instrument's ability to identify gang presence and also show that our instrument is not significantly correlated with residents’ general fear of crime or their view of general crime and violence.

To explore the mechanisms underpinning the emergence of criminal governance in a large urban setting, we move the analysis from the gang level to the community level. For the purposes of this work, we define a community using the Middle layer Super Output Areas (MSOAs) created by the UK Office for National Statistics (ONS). Greater London can be subdivided into 982 MSOAs, and each of these census units includes an average of 8316 individuals. MSOAs have two desirable properties: (a) they have a geographical component, that is, they are designed taking into account geographical barriers, for example, a river, rail tracks or main roads; hence, they can approximate spatialised community-level social interactions; and (b) they show stability over time (contrary to, e.g., electoral wards). We asked the questionnaire respondents to place each gang within a spatially defined area based on the postcode of the ‘centre of gravity’ of a gang's activity and then linked each postcode to the corresponding MSOA (UK postcodes are perfectly nested within MSOAs). In this work, we use the terms community, neighbourhood and MSOA interchangeably.

We now turn to discuss the dependent variable and the set of independent and control variables included in our analysis.

### Dependent variable

Our *dependent variable* is a categorical variable capturing the type of gang presence in a community (MSOA). Based on the responses to the Crim-Gov questionnaire, we identified three types of communities: (a) those with no gang presence; (b) those with gang presence but no criminal governance; and (c) those with criminal governance.^3^

**Figure 1.**
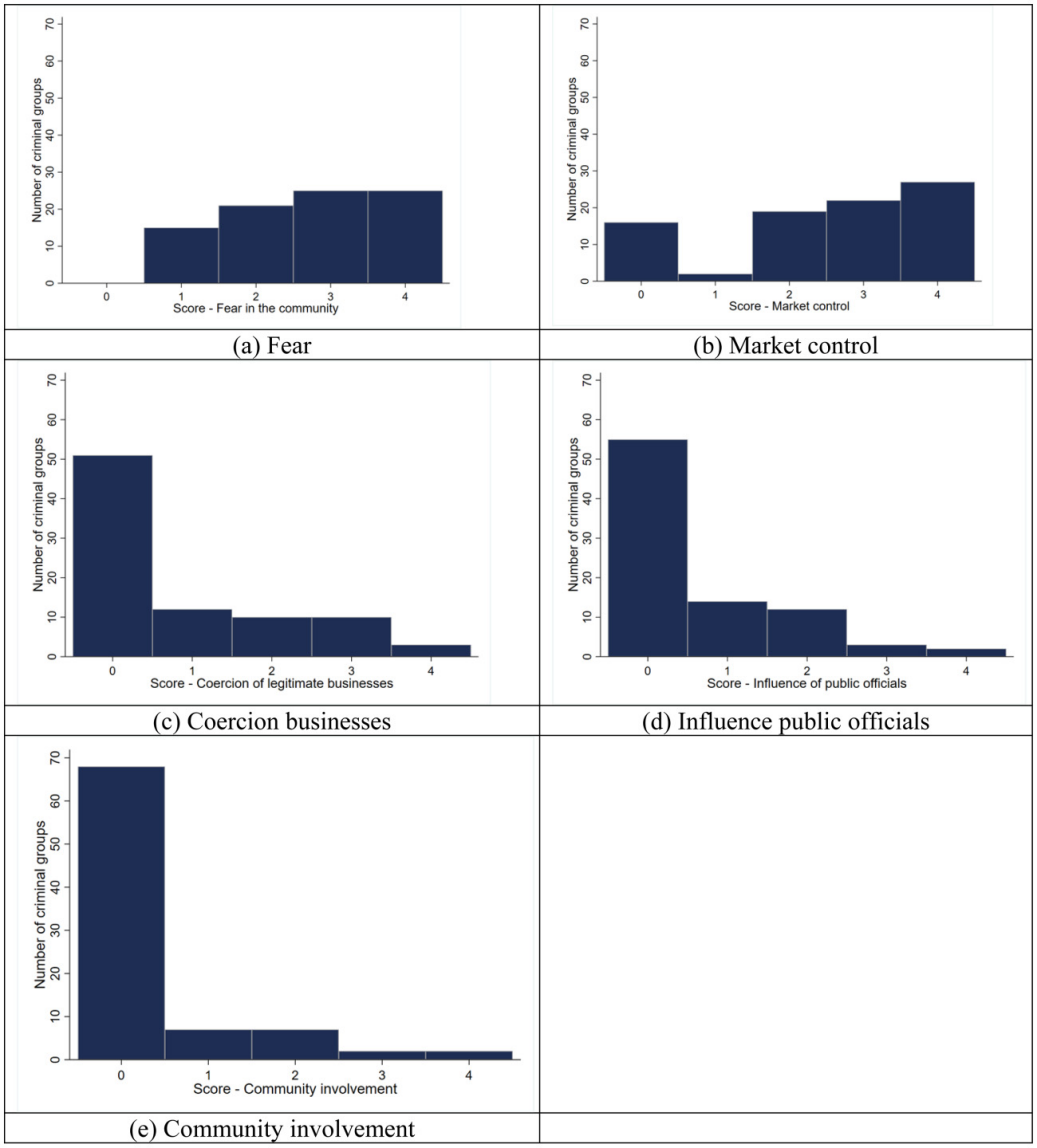
Crim-Gov questionnaire: distribution of gangs’ scores by activity (*N* = 86).

As mentioned above, gangs can control illegal markets only, such as drugs, or be involved in legal markets and community activities too. In this paper, we are most interested in identifying gangs that are able to make their presence felt in legal markets and in the community at large. Such criminal groups pose the greatest threat to the legitimacy of authorities, hence deserve special attention. For this reason, we adopt a strict definition of criminal governance, including only those gangs able to exert some degree of governance beyond just illegal markets, that is, impacting legal businesses, public officials and broader community activities. Thus, we define gangs as involved in criminal governance activities if they engage in at least one of the following activities: coercion of legitimate businesses; influence of public officials; involvement in community activities. While such questions do not imply that gangs have total control over such spheres of life, they do indicate the gangs’ ability to reach there. The next decision we took relates to scoring. Although the questionnaire includes five values (from 0 to 4) and thus allows us to capture the intensity of an activity, we decided to dichotomise the data for the models developed below due to statistical reasons; however, future studies might wish to go beyond such a limitation. The distribution of scores for each dimension is presented in Figure 1. In conclusion, we consider as ‘governance-type’ those gangs that have received a score of 1 or higher in at least one among questions 2 (coercion of legitimate businesses), 3 (influence of public officials) and 5 (involvement in community activities) of the Crim-Gov questionnaire.

### Independent variables

To explore the emergence of governance, we consider several *independent variables*, measuring the social, demographic, and economic characteristics of 982 communities (MSOAs) across Greater London. The variables are drawn from a variety of ONS sources (see Table A2 in Appendix 3) and measured at the MSOA level. We consider values for the year closer to the Crim-Gov survey (2019). While most variables were measured in 2019 or the years shortly before (see Table A2 in Appendix 3), variables based on the UK census are the exception: as results from the 2021 census were not yet available at the time of data collection and analysis, we had to rely on data from the 2011 census. However, we believe that this has a limited impact on the analysis as such variables tend to refer to structural features of a community that are normally slow-changing, such as educational levels or age structure.

The independent variables included in our econometric model are socioeconomic disadvantage, accessibility to local services, residential mobility, and migrant concentration (Table 1 reports summary statistics for such variables). To measure *socioeconomic disadvantage* of a community, we consider four alternative measures: (1) an index of income deprivation based on the proportion of population in a given community experiencing deprivation due to low income as the result of either unemployment or low earnings; (2) household income, measuring the average annual income of households in a given community; (3) a variable measuring the proportion of residents aged 16 and over with no educational qualifications to account for disadvantage in the educational domain; (4) an index of health deprivation, which measures the risk of premature death and impairment to the quality of life due to poor physical and mental health. We consider these multiple measures of socioeconomic disadvantage in separate model specifications to avoid issues of multicollinearity.^4^

**Table 1.** Summary statistics of independent variables included in the MSOA econometric model.

Variable	Mean	Std. Dev.	Min	Max	*N*
Population density	83.48	47.99	2.90	247.20	982
Population aged 15–24 (%)	13.44	3.39	6.10	41.60	982
Income deprivation (%)	13.62	6.20	0.94	33.49	982
Household income (£; scaled/1000)	53.53	8.57	36.00	85.20	982
Lack of education qualifications (%)	17.79	6.36	3.60	37.70	982
Health deprivation (score)	26.62	5.99	0.00	40.04	982
Barriers to housing/services (score)	31.74	8.63	9.52	59.92	982
5-year residential mobility (%)	17.18	5.30	5.55	45.26	982
% born outside the United Kingdom	36.21	13.84	4.39	69.23	982

*Note:* MSOA: Middle layer Super Output Area; Std. Dev.: standard deviation; Min: minimum; Max: maximum.

As a measure of *accessibility to local services*, we consider an index developed by the ONS and included in the ‘Barriers to housing and services’ domain of the Index of Multiple Deprivation. This index measures the physical and financial accessibility of local services and housing. It is computed by combining (with equal weights) indicators pertaining to two sub-domains: the ‘geographical barriers’ sub-domain, which relate to the physical proximity of local services, and the ‘wider barriers’ sub-domain, which includes issues relating to access to housing, including social housing (McLennan et al., 2019). The geographical barriers sub-domain includes indicators measuring the average geographical proximity of a range of local services (e.g., post office, primary school, general practitioner (GP) surgery). The indicators included in the wider barriers sub-domain measure household overcrowding, the homelessness rate, and housing affordability. We consider the barriers to services and housing as a proxy for the level (and to some extent effectiveness) of services provided by local and central authorities.

*Residential mobility* is calculated as the proportion of resident households that have changed within a specific time period (5 years in our analysis). *Migrants’ concentration* is measured by the proportion of community residents not born in the United Kingdom. Finally, all model specifications include the *population density* and the *proportion of population aged 15–24*. The latter captures the (relative) size of the more crime-prone segment of the population.

### Analytic strategy

We first present results on the distribution of gangs and criminal governance across London communities. We then rely on a multinomial logistic regression model to examine the factors underpinning the emergence of gang-related governance in a given community. This modelling approach is particularly suitable when predicting more than two outcomes given a set of independent variables and with unordered outcome categories (Britt and Weisburd, 2010; Tabachnick and Fidell, 2013). This model is an extension of a binary logistic regression model that simultaneously accounts for changes in the odds of multiple outcomes following changes in the value of the independent variables (Britt and Weisburd, 2010). The model estimated in this paper is defined as follows:

$$\log\left(\frac{\pi_{m}(x)}{\pi_{M}(x)}\right)=\alpha_{m}+\beta_{m}^{\prime }x,\;\;m=1,\ldots ,\;M-1$$

where x is the set of independent variables included in the analysis and $\pi_{m}(x)=P(Y=m|x)$ . In our case, M=3 since the dependent variable has three outcome categories: ‘no gang presence’, ‘gang presence with no criminal governance’, and ‘gang presence with criminal governance’. We consider the absence of gangs as the reference category: the set of coefficients predicting each outcome can thus be interpreted as the log of the odds (odds ratio) comparing the likelihood of that specific outcome (e.g., gang presence with no criminal governance) relative to observing no gang presence in the community.

We run a set of models with different specifications aiming to understand how different measures of deprivation, accessibility to local services, migrants’ concentration, 5-year residential mobility, population density and the share of the population aged 15–24 impact the presence of gangs, separating those involved in criminal governance from those not inolvolved in criminal governance.

When presenting and discussing our results, we are careful to avoid the use of causal language. In other words, we cannot completely exclude reverse causality for a number of variables we input as independent in the multinomial logistic regression (see Table 3). Yet it is hard to imagine that the presence of a gang would have a causal effect, for example, on barriers to housing and services or the percentage of individuals born outside the United Kingdom. Interestingly, Booth's (1889) London maps from 1889 contain information on income indicators: London areas’ income strongly correlates with current data, although Booth's maps only cover Inner London (analysis available on request). However, as our econometric setup does not allow us to exclude that some of the variables we analyse are affected by a gang's presence, our results should be interpreted as showing the existence (or absence) of a relation between relevant factors and gangs.

## Criminal governance in London communities

The Crim-Gov survey has identified 86 gangs operating across London. Their level of involvement in criminal governance-related activities varies, as shown in Figure 1(a–e).

Two findings are of particular interest. Firstly, while all gangs generate fear in the communities in which they operate – and 58% of them generate strong fear, that is, a score of 3 or 4 – not all are involved in the three key dimensions of governance: coercion of legal businesses, influence of public officials and involvement in community activities. In fact, only 51 gangs (60%) are involved in at least one of these three activities; this number goes down to 16 (19%) if we consider gangs that have a strong involvement in at least one of these activities. This suggests that moving from just being a fear-generating gang to a more complex organisation involved in criminal governance is not an easy task – and is a step that only a fraction of gangs is able to make.

Secondly, 81% of gangs are able to exert some degree of control over the criminal market in which they operate – most likely a drug market, although we have no first-hand evidence – and 57% are able to exert strong control. These are a much larger proportion than the gangs able to provide governance impacting legitimate businesses, public officials and the broader community. This suggests that providing criminal governance beyond illegal markets requires a more complex set of resources and skills that fewer gangs actually possess.

When placing gangs within spatially defined communities, we observe gang presence in 79 communities (out of 982; i.e., 8.75% of all MSOAs): 33 communities are affected by non-governance gangs, while 46 experience some degree of criminal governance (4.7% of all MSOAs; Table 2). In 20 communities (around 25% of those with gang presence), gangs engage in minor to moderate attempts of coercion of illegal businesses (e.g., threats and extortion directed to business owners), while in 13 communities (roughly 16%) coercion is reported to be either strong or very strong. In 28 communities across London, gangs are able to exert some degree of influence on public officials, and in five communities, gangs’ influence has been qualified by police officers as strong. Involvement of criminal groups in community activities – ranging from the offer of assistance to residents to dispute settlement – has been reported in 17 areas, and in four it is considered either strong or very strong.

**Table 2.** Gang presence and criminal governance in London communities: mean component scores.

	No gang presence	Gang presence	Gang presence and criminal governance
Fear in the community	0	2.27 (1.10)	3.09 (0.86)
Market control	0	1.61 (1.46)	3.20 (0.93)
Coercion of legitimate businesses	0	0	1.54 (1.29)
Influence of public officials	0	0	1.09 (1.13)
Community involvement	0	0	0.72 (1.13)
*N*	903	33	46

*Note:* Standard deviations are reported in parenthesis.

There are four communities across London in which gangs are reported to engage in all three key criminal governance dimensions of coercion of legitimate businesses, influence of public officials, and involvement in community activities. In roughly half of communities with criminal governance (24 out of 46), gangs present two of these three sets of activities; in 18 communities, they are reported to engage in one set only.

Gangs providing forms of criminal governance in their community generate on average a higher level of fear in the same community and have a higher level of control of the markets they are involved in (both differences in means are statistically significant at .01 level; see Table 2). Overall, average scores for governance activities are smaller than those for fear and market control, thus pointing to a more complex criminal endeavour.

**Figure 2.**
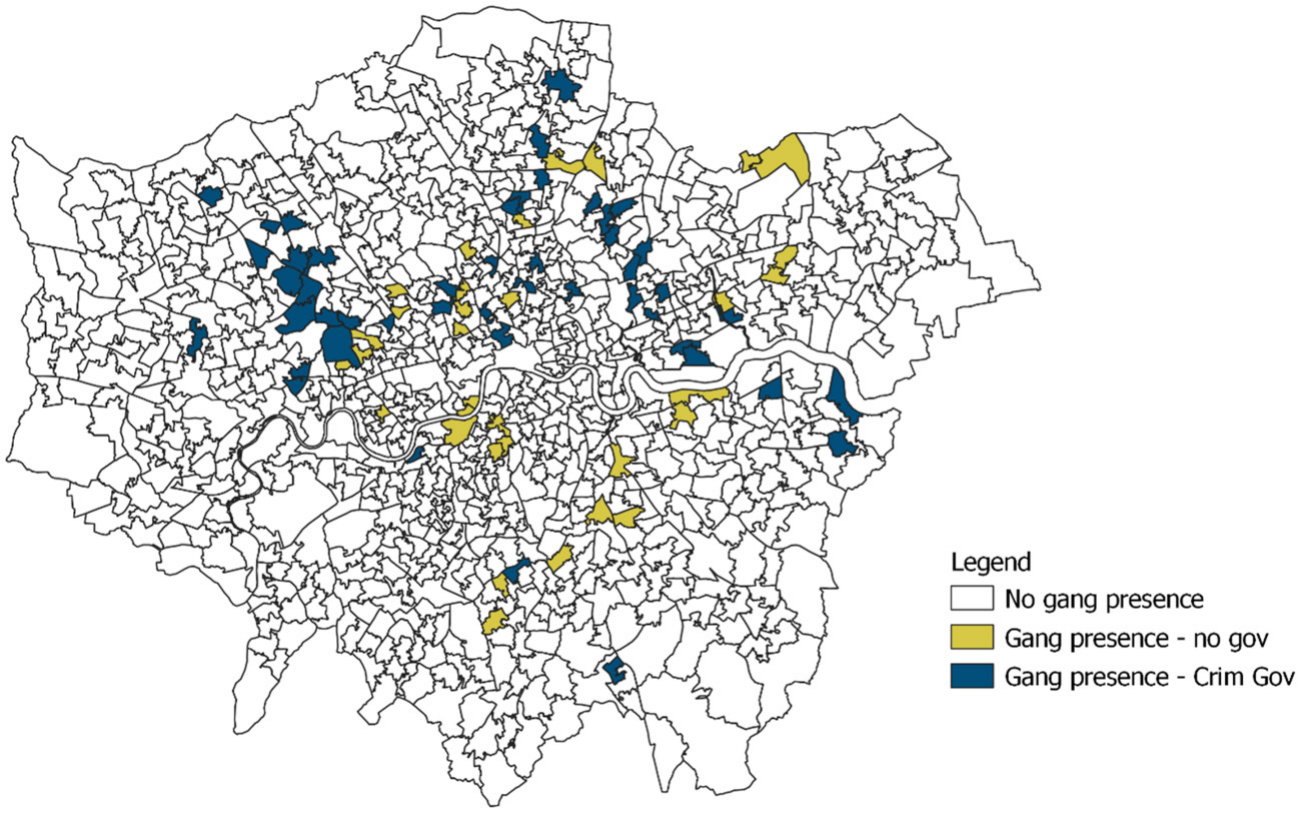
Spatial distribution of gangs and criminal governance.

Figure 2 presents the geographic distribution of the three sets of communities identified in this analysis. Gang presence is recorded across the whole city, and in most boroughs, there is at least one gang operating. Criminal governance appears to be relatively more clustered, affecting particularly the North Eastern and North Western communities. The boroughs of Brent, Islington, Newham, and Waltham Forest are particularly impacted, with more than 10% of their MSOAs recording the presence of a gang providing some form of governance. Brent emerges as the borough most affected, with almost 30% of communities (10 out of 24 MSOAs) registering the presence of gang-related governance.

In sum, criminal governance appears to be highly concentrated in a few areas – and not all areas with a gang presence also experience criminal governance. What makes a community more vulnerable to criminal governance, then? We now turn to this question.

## The emergence of criminal governance

To explore the factors underpinning criminal governance in communities, we test several multinomial logistic regression models. As a reminder, in this work we define a community as affected by criminal governance if the gang(s) operating in the area engage in at least one of the following three activities: coercion of legitimate businesses, influence of public officials or involvement in community activities. We separate these communities from those in which gangs are present but do not engage in criminal governance and from communities where no gang presence has been identified through our survey.

Table 3 presents the results of the first set of models exploring the impact of different measures of deprivation on the probability of criminal governance. In all models, an increase in the level of deprivation is related to an increase in the odds of gang presence; all other variables held constant – a result also found in other studies (e.g., Kirchmaier, Machin, Villa-Llera, 2020: 9–10). This finding is robust to different deprivation measures: income deprivation index, total household income, education deprivation, and health deprivation index. For example, an increase of one percentage point in the proportion of residents experiencing low-income deprivation is associated with an increase by a factor of 1.22 in the relative risk of a community recording a non-governance gang compared to an increase of 1.15 for a governance gang. Similarly, an increase of £1000 in the average annual income of households residing in the community decreases the relative risk of non-governance gang by a factor of 0.85 and of 0.88 for a criminal governance gang. While the coefficients for deprivation are higher for non-governance gangs compared to criminal governance ones across all models, the difference in magnitude is not statistically significant in any of the models (results from a Wald test for the equality of coefficients).

**Table 3.** Community characteristics and criminal governance: multinomial logistic regressions (relative-risk ratios).

	Reference category: MSOAs with no gang presence							
	(1)	(1)	(2)	(2)	(3)	(3)	(4)	(4)
	Gang No gov	Gang Crim-Gov	Gang No gov	Gang Crim-Gov	Gang No gov	Gang Crim-Gov	Gang No gov	Gang Crim-Gov
Population density	1.004	1.010*	1.008	1.012**	1.011*	1.014***	1.006	1.011**
	(0.005)	(0.004)	(0.005)	(0.004)	(0.005)	(0.004)	(0.005)	(0.004)
Population aged 15–24 (%)	0.961	0.999	0.912	0.950	0.973	0.989	0.940	0.968
	(0.065)	(0.060)	(0.061)	(0.059)	(0.066)	(0.066)	(0.064)	(0.062)
Income deprivation	1.217***	1.152***						
	(0.045)	(0.036)						
Household income			0.849***	0.884***				
			(0.032)	(0.031)				
Lack of education qualifications					1.116**	1.110**		
					(0.040)	(0.038)		
Health deprivation							1.309***	1.257***
							(0.075)	(0.064)
Barriers to housing/services	0.954	1.065*	0.947	1.056*	0.979	1.071**	0.961	1.068**
	(0.029)	(0.026)	(0.029)	(0.027)	(0.029)	(0.027)	(0.029)	(0.026)
5-year residential mobility	0.968	0.861**	1.036	0.892*	1.010	0.891*	0.877*	0.786***
	(0.048)	(0.041)	(0.054)	(0.043)	(0.049)	(0.042)	(0.049)	(0.043)
% born outside the United Kingdom	1.013	1.041*	1.011	1.034*	1.020	1.042*	1.049*	1.071***
	(0.021)	(0.019)	(0.020)	(0.018)	(0.021)	(0.019)	(0.024)	(0.022)
Constant	0.009***	0.001***	492.4*	4.423	0.001***	0.000***	0.000***	0.000***
	(0.011)	(0.001)	(1334.732)	(11.086)	(0.002)	(0.000)	(0.000)	(0.000)
Observations	982	982	982	982	982	982	982	982

*Note:* Standard errors are reported in parenthesis. Household income variable was scaled by dividing it by 1000 to ease interpretability. MSOAs: Middle layer Super Output Areas.

The symbols ***, **, and * indicate statistical significance at the .001, .01, and .05 levels, respectively.

The level of existing barriers to services and housing is positively associated with the odds of criminal governance (compared to no gang presence), while it is unrelated to the odds of gang presence with no governance. This is a key result which indicates that, controlling for all the other variables, communities where residents face greater difficulties in access to services see a higher probability of criminal governance in their territory. This variable can be interpreted as a proxy for the level (and to some extent the effectiveness) of services provided by local and central authorities; our result thus suggests that criminal governance is more likely to emerge where legal governance is of lesser quality. Crucially, this does not extend to gang presence in general, suggesting a profound difference in the nature of criminal governance vis-à-vis other gang activities.

Furthermore, an increase in the proportion of community residents born outside the United Kingdom is related to increased odds of criminal governance in the community (compared to no gang presence). Holding all the other variables constant, results show an increase by a factor of 1.03–1.07 in the relative risk of criminal governance for a one percentage point increase in the proportion of residents born outside the United Kingdom. This result is robust to the inclusion of different deprivation measures in the models. Conversely, the concentration of migrants tends not to be significantly associated with the odds of gang presence with no criminal governance (with the exception of model specification 4). The proportion of the population aged 15–24 is unrelated to both gang presence and criminal governance.

Finally, as for the residential mobility of an area, we find that the 5-year residential mobility is negatively associated with criminal governance. Holding the other variables constant, more stable communities, that is, those with a lower turnover of resident households over a 5-year time, face higher odds of gang-related criminal governance. Therefore, it is not the areas with the highest turnover of residents, and perhaps the weakest social fabric, that experience the most serious type of gang presence.

## Discussion and conclusions

This study makes three significant contributions. The first is methodological in nature. We introduce a novel survey instrument – the Crim-Gov questionnaire – that allows us to identify key defining dimensions of a criminal group: its ability to generate fear in the community, coerce legitimate businesses, influence public officials, control markets and be involved in community activities. The questionnaire does not require a long time to complete; hence, it can be administered to busy officials and experts in a highly cost-effective manner. Rather than relying on non-systematic newspaper reports and web stories (cf. Kirchmaier et al., 2020), we have used a systematic method of data collection.

The second contribution is descriptive. We have been able to identify instances of governance-related activities among the gangs operating in London. We show that providing criminal governance, particularly beyond illegal markets, is a complex task requiring a set of resources and skills that fewer gangs possess. When looking at communities, criminal governance appears to be highly concentrated in a few areas – and not all areas with a gang presence also experience criminal governance. We document that 46 communities (out of 79 with gang presence) experience some degree of criminal governance; in 17 communities, gangs are involved in community activities, while in four communities gangs engage in all three dimensions of governance (coercion of legitimate businesses/influence of public officials/community involvement).

Third, we test several models that explore the relationship between socioeconomic disadvantage, accessibility to local services, residential mobility, migrant concentration, and gangs. In line with results from large US cities (Curry and Spergel, 1988; Papachristos and Kirk, 2006; Pyrooz et al., 2010; Katz and Schnebly, 2011; see also Decker et al., 2022: Ch. 2), also in the case of London we find that the level of deprivation is related to an increase in the odds of gang presence. Furthermore, we find that this effect holds across *all* types of gang presence, governance and non-governance ones. On the other hand, we find a differential impact of access to services on governance and non-governance gangs. Crucially, greater difficulties in accessing local services and housing are *only* related to a higher probability of criminal governance (the same does not hold for non-governance gangs). We conclude that such a phenomenon is more likely to emerge where the legal provision of services is of lesser quality. We have pointed out above that criminal governance should be thought of as a continuum: in some settings, criminal groups can substitute key state functions, as documented in traditional mafia areas (see e.g., Sabetti, 1984; Gambetta, 1993; Varese, 2011). Our work shows that explanations invoked for such cases also apply to instances where criminal governance is less pervasive. These findings suggest the need for a unified theory of criminal governance that encompasses mafias and ‘cartels’ at one end and urban gangs at the other.

We further find that the 5-year residential mobility is negatively associated with criminal governance, suggesting that it is not the areas with the weakest social fabric that experience the most serious type of gang presence, but those with a more stable residential population. A certain degree of stability is needed for criminal governance to emerge, for example to allow gangs to collect and circulate information more efficiently within the community. These results contribute to previous findings on the negative association between residential mobility and gang presence (Wells and Weisheit, 2001; Katz and Schnebly, 2011) by pinpointing how this association might be driven by governance activities. Finally, an increase in the proportion of residents born outside the United Kingdom is consistently associated with higher odds of criminal governance in the community across all models (while the relationship with non-governance gangs is more ambiguous).

Quantitative work on gangs and organised crime is difficult and subject to limitations; our study is no exception. While we are reassured by the level of external validity shown by our survey instrument (see Appendix 2), limitations stemming from the nature of police evidence remain, and future works might wish to apply our instrument to non-police officials and local stakeholders to get a more comprehensive view of the phenomenon. In addition, our work is based on one snapshot of the London gang landscape; however, such landscape is likely to change over time, and future works might benefit from including a longitudinal design.

Finally, our work suggests two main policy implications. Firstly, it is crucial for law enforcement – and local stakeholders – to not only collect data on gangs but also on the type of activity such groups are involved in. We strongly recommend that particular attention is devoted to identifying instances of criminal governance and to treating such gangs separately from the non-governance ones. While more concentrated in space and less prevalent in scale, criminal governance generates *systemic* harm to cities and countries, creating lasting damages to the institutional and social environments of places and communities alike. It is arguably more difficult to eradicate if left unchecked. Secondly, our work has shown that interventions targeting socio-economic factors might not be sufficient either. When tackling the emergence and presence of governance-type gangs, policy-makers should also improve access to and quality of public services. This is a mechanism that does not always receive the attention it deserves.

## Notes

1. The questionnaire expands on the three-item Crim-Gov questionnaire first presented in Campana and Varese (2018). This novel iteration of the instrument adds the two dimensions of market control (question 4) and community involvement (question 5) besides a relabelling of the scales. Such changes enable us to capture dimensions suggested by Lessing (2021) who unpacks the phenomenon into five different categories: (1) policing, (2) judicial, (3) fiscal, (4) regulatory, and (5) political. The practices captured by Lessing (2021) include surveillance, enforcement of property rights, dispute resolution, the fulfilment of contractual agreements, tax collection, the provision of credits and involvement in community or electoral politics.

2. As a reference, the Metropolitan Police defines a gang as a ‘relatively durable, predominantly street-based group of young people who (1) see themselves (and are seen by others) as a discernible group and (2) engage in a range of criminal activity and violence. They might also have any or all of the following features: identify with or lay claim over territory; have some form of identifying structure feature; are in conflict with other, similar gangs’ (see: https://www.met.police.uk/police-forces/metropolitan-police/areas/about-us/about-the-met/gangs-violence-matrix/).

3. In most communities with gang presence, there is only one active gang. Only six MSOAs register more than one gang; in these cases, we assigned to the MSOA the highest score recorded among the gangs active in the area.

4. As expected, these measures show a relatively high level of correlation: minimum pairwise correlation |*r*|=.52, maximum |*r*|=.81.

5. This source of data has already been utilised by criminologists to explore crime and police-related attitudes. See Bradford et al. (2009).

## Appendix 1. Crim-Gov questionnaire

**Q1: On a scale from 0 to 4, how much fear does this group generate in the community?** (e.g., the group engages in disrespectful behaviour, threatens members of the public, resorts to violence, and generates a ‘no grass’ culture in the community)

⚪   ⚪  ⚪  ⚪  ⚪

01  2  3  4

No Fear        Very strong fear

**Q2: On a scale from 0 to 4, to what extent does this group coerce legitimate businesses?** (e.g., the group threatens business owners, engages in extortion, and influences business decisions)

⚪  ⚪  ⚪  ⚪  ⚪

0  1  2  3  4

No Coercion     Very strong coercion

**Q3: On a scale from 0 to 4, to what extent does this group influence public officials?** (e.g., the group influences probation officers and other officials involved in its case file, influences local officials’ decisions, influences local councillors and politicians)

⚪  ⚪  ⚪  ⚪  ⚪

0 1  2 3 4

No Influence Very strong influence

**Q4: On a scale from 0 to 4, to what extent does this group control the market(s) it is involved in?** (e.g., the members sell illegal products, they decide who is allowed to sell illegal products, they successfully keep rival groups away from their territory)

⚪  ⚪  ⚪  ⚪  ⚪

0  1  2 3 4

No Control      Very strong control

**Q5: On a scale from 0 to 4, to what extent is the group involved in community activities?** (e.g., the group offers assistance to neighbourhood residents and settles disputes between community members)

⚪  ⚪  ⚪  ⚪  ⚪

0 1 2 3 4

No Involvement    Very strong involvement

**Q6: Please provide a full postcode for the best-estimated centre of the group’s territory.**

## Appendix 2. External validity of Crim-Gov questionnaire

To assess the external validity of our survey instrument (Crim-Gov questionnaire), we leverage data from the 2016–17 MOPAC (Mayor of London – Office for Policing and Crime) Public Attitudes Survey (ORS, 2017). The survey was conducted between October 2016 and September 2017 on a total of 12,800 respondents and is based on a random sample stratified by BCU with a target of 400 individuals per borough (further information is included in ORS, 2017). Due to limited sample size and unavailability of personal information on the MSOA of respondents, the analysis could only be conducted at the electoral ward level, which is a slightly larger spatial unit than the MSOA (average population of 13,171 in Greater London).^5^

**Table A1.** Attitudes to local crime problems and Crim-Gov survey variables: correlation table.

	1	2	3	4	5
1. Worried about crime	1				
2. General crime is a problem	0.60*	1			
3. Violence is a problem	0.44*	0.56*	1		
4. Gangs are a problem	0.45*	0.30*	0.45*	1	
5. Gang presence (total gang score from Crim-Gov survey)	0.04	−0.03	0.07	0.19*	1

*Note:* The symbol * indicates that the correlation is statistically significant (at 0.05 level).

In Table A1, we compare residents’ answers on gang presence (‘gangs are a problem’) with our results on the strength of gang presence (measured as the sum of the scores of all five survey dimensions) at the ward level. The two variables are positively and significantly correlated, thus corroborating our instrument’s ability to identify gang presence. Crim-Gov then goes a step further in sorting gangs based on their ability to provide criminal governance. Moreover, our instrument is not significantly correlated with residents’ general fear of crime or their view of general crime and violence as major issues in the community. This further points to the ability of our survey instrument to identify gang presence and not general crime.

## Appendix 3

**Table A2.** Independent variables included in the econometric analysis.

Variable label	Description	Source (year)
Population density	Number of resident population per hectare.	Census for England and Wales (2011)
Population aged 15–24 (%)	Proportion of the total population aged between 15 and 24.	Census for England and Wales (2011)
Income deprivation	Score of the ‘Income deprivation’ domain of the Index of Multiple Deprivation (higher score corresponds to higher deprivation); measures the proportion of the population experiencing deprivation related to low income.	Index of Multiple Deprivation, ONS (2019)
Household income (scaled /1000)	Average annual household income (£), financial year ending 2018. In the models, it has been scaled by dividing it by 1,000 to ease coefficient comparability.	ONS (2018)
Lack of education qualifications	Proportion of residents aged 16 and over with no education qualifications.	Census for England and Wales (2011)
Health deprivation	Score of the ‘Health deprivation’ domain of the Index of Multiple Deprivation (higher score corresponds to higher deprivation); measures the risk of premature death and the impairment of quality of life through poor physical or mental health.	Index of Multiple Deprivation, ONS (2019)
Barriers to housing/services	Score of the ‘Barriers to housing and services’ domain of the Index of Multiple Deprivation (higher score corresponds to higher deprivation); measures the physical and financial accessibility of housing and local services (physical proximity of local services and issues relating to access to housing such as affordability and homelessness).	Index of Multiple Deprivation, ONS (2019)
5-year residential mobility	Estimate of the ‘churn’ of the residential population: the proportion of households that have changed between the end of 2020 and five years prior.	CDRC Residential Mobility Index (2020)
% born outside the UK	Proportion of the resident population not born in the UK.	Census for England and Wales (2011)

*Note:* Variables collected from the 2019 Index of Multiple Deprivation were produced at the LSOA level and were aggregated to the MSOA level by summing the population-weighted scores of the corresponding LSOAs.
